# Mitigation of Quantum Dot Cytotoxicity by Microencapsulation

**DOI:** 10.1371/journal.pone.0022079

**Published:** 2011-07-21

**Authors:** Amelia Romoser, Dustin Ritter, Ravish Majitha, Kenith E. Meissner, Michael McShane, Christie M. Sayes

**Affiliations:** 1 Interdisciplinary Program of Toxicology, Texas A&M University, College Station, Texas, United States of America; 2 Department of Biomedical Engineering, Texas A&M University, College Station, Texas, United States of America; 3 Materials Science & Engineering Program, Texas A&M University, College Station, Texas, United States of America; University of Milano-Bicocca, Italy

## Abstract

When CdSe/ZnS-polyethyleneimine (PEI) quantum dots (QDs) are microencapsulated in polymeric microcapsules, human fibroblasts are protected from acute cytotoxic effects. Differences in cellular morphology, uptake, and viability were assessed after treatment with either microencapsulated or unencapsulated dots. Specifically, QDs contained in microcapsules terminated with polyethylene glycol (PEG) mitigate contact with and uptake by cells, thus providing a tool to retain particle luminescence for applications such as extracellular sensing and imaging. The microcapsule serves as the “first line of defense” for containing the QDs. This enables the individual QD coating to be designed primarily to enhance the function of the biosensor.

## Introduction

Nanoscale materials are promising contenders for diagnostics, therapeutics, and imaging agents due to their size, functionality, and unique optical properties. Many of the proposed biomedical applications for nanomaterials revolve around their employment as targeted drug delivery vehicles in the circulatory system [1], [2]. Another potential biomedical application of nanomaterials includes their incorporation into medical implants, such as devices placed in the subcutaneous tissue or even as functional elements of “smart” tattoo-like biosensors [3], [4], [5]. Such concepts require engineered structures and materials with the desired function (e.g., optical sensing) within a fully biocompatible and/or biodegradable platform [5]. As an example, many biosensors require mobility of sensing reagents—the sensors/reagents must be able to, allowing them to freely associate and dissociate, while being physically constrained to enable continuous use in one location [5], [6]. Inorganic nanoparticles offer unique properties that enable innovative biosensing techniques. However, it is difficult to localize the nanoparticles for long periods of time. Additionally, the prospective use of nano-enabled biosensors for such *in vivo* applications has raised concerns regarding the possible localized and systemic toxicological effects in humans. Mechanistic analyses of these effects in humans are needed when assessing the risks due to the use of nanomaterials in medicine and biological imaging.

QDs with a cadmium selenide core (CdSe) and a zinc sulfide (ZnS) shell remain the most studied, produced, and proposed luminescent nanomaterial. Offering significant advantages for energy transfer-based biosensors, QDs are photobleaching resistant, have high quantum yield, and possess broad absorption/narrow emission bands that are size tunable [17]. However, since CdSe/ZnS QDs have been shown to enter living cells [7], [8], [9], [10], [11], [12], their toxicological characterization and mitigation is extremely relevant to nanobiotechnology. It has been shown that the addition of a ZnS outer shell can minimize damage to the cell [13]; however, the potential for substantial damage from leaching cadmium, selenium, and/or excess zinc still exists [14], [15]. In addition, because QDs are intrinsically redox-active, a portion of their toxic potential may also arise from such native properties without regard to their composition, surface properties, or cellular internalization potential. Quantum dots can transfer absorbed optical energy to adjacent oxygen molecules, thus spontaneously generating reactive oxygen species (ROS) such as hydroxyl radical (^•^OH), superoxide (O^2−^), and singlet oxygen (^1^O_2_) [16], [17], [18]. Further modification of the QD surface with silanes [19], oligomeric phosphines [20], phospholipids [21], and amphiphilic triblock copolymers [22] has been demonstrated as an effective means to further mitigate toxicity by protecting the QD surface from deterioration in biological media. However, these capping agents increase overall QD size enough to preclude efficient energy transfer to an acceptor [23]. Therefore, although these bulky capping agents protect the QD from degradation, biosensing schemes requiring intimate contact between QDs and analytes/reagents (e.g., transduction via energy transfer) can incur a loss in biosensor functionality.

As an alternative to protecting individual QDs, microencapsulation provides a means to modulate interfacial interactions between the cells and QDs without the need to deposit bulky surface coatings on the QDs. Our work is separate from a large body of work focused on encapsulating individual QDs, as we are microencapsulating an ensemble of QDs (2.05e10) within each polyelectrolyte microcapsule (2.05e10 QDs/microcapsule). It is also noteworthy that the QDs used in this study are microencapsulated within the hollow interior (i.e., void) volume of the polyelectrolyte microcapsule, which should be distinguished from QD entrapment within the polyelectrolyte film itself [24] and results in an interaction among the QDs, the solvent, and other molecules that permeate the film. Although the two seem similar superficially, important differences exist with respect to interactions with surroundings and apparently toxicity, as evidenced by our data.

**Figure 1:**
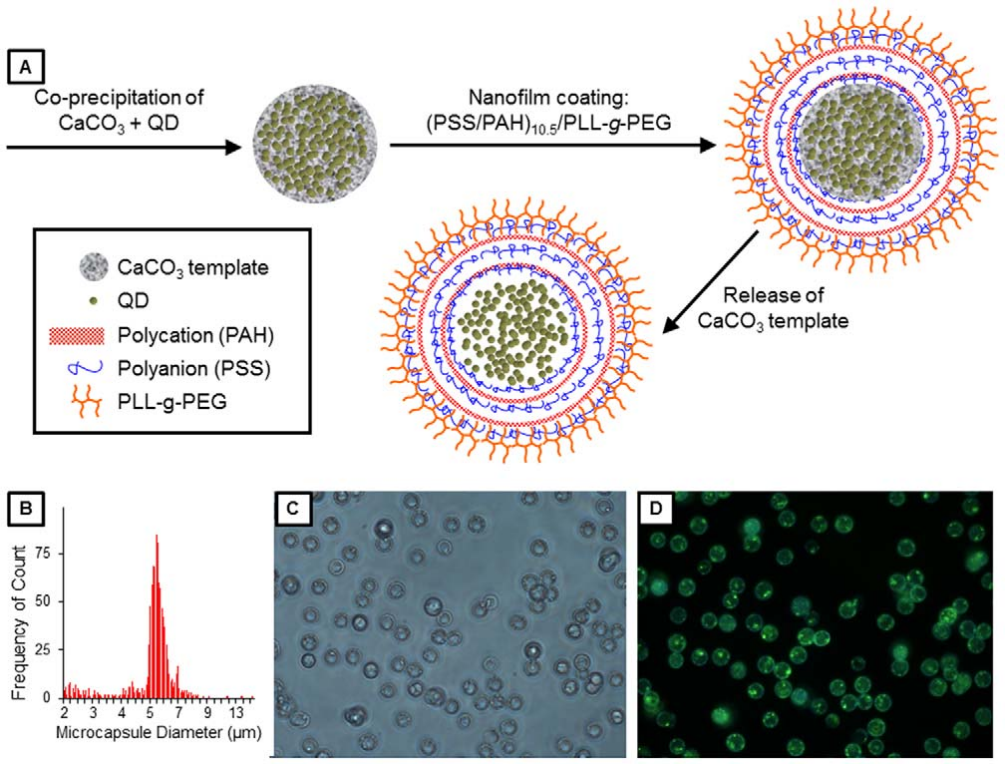
Quantum dot-loaded microcapsule characterization. (A) Schematic representation of the formation of QD-loaded microcapsules. (B) Histogram of the size distribution of QDMCs. (C) Brightfield and (D) fluorescence images of the QDMCs, showing that the microcapsules are monodisperse and contain QDs (ex/em 380/545).

Microcapsules and nanoparticle surface coatings can function as protective layers to prevent or inhibit erosion, oxidation, and leaching of core components; however, the leaching of core components is a central challenge for the microencapsulation field [25]. Numerous studies have shown that microcapsules synthesized via the layer-by-layer (LbL) process inhibit the release of core components and show promise for the development of new types of nano-enabled biosensors [25], [26], [27]. Even one of the smallest enzymes, trypsin (M_w_ = 23.3 kDa; corresponding to an average diameter of ∼1–3 nm) does not leach from an LbL microcapsule [28]. The PEI-coated QD materials (termed CdSe/ZnS-PEI) used in this study are ∼15 nm in all dimensions and are thus well contained within the microcapsule (Figure 1). However, it is important to note that the permeability of the microcapsules to ions, small molecules, and even macromolecules can be tailored in a number of ways, including modulation of the pH (during and post-construction), ionic strength (during and post-construction), solvent (during and post-construction), polymer composition, and shell thickness (Antipov and Sukhorukov, 2004). Therefore, given a sufficiently large disparity in molecular weight/size between the analyte and the QD (or other assay components), a number of strategies can be employed to entrap the sensing assay, while maintaining permeability of ions and small molecules (e.g., the analyte). Again, because the polyelectrolyte microcapsules are hollow, the QDs retain their responsiveness to the environment. Therefore, the purpose of this study was to examine the protective effects of additional surface constructs, namely polymer microcapsules, in comparison with the toxicological effects of free CdSe/ZnS-PEI QDs.

**Figure 2:**
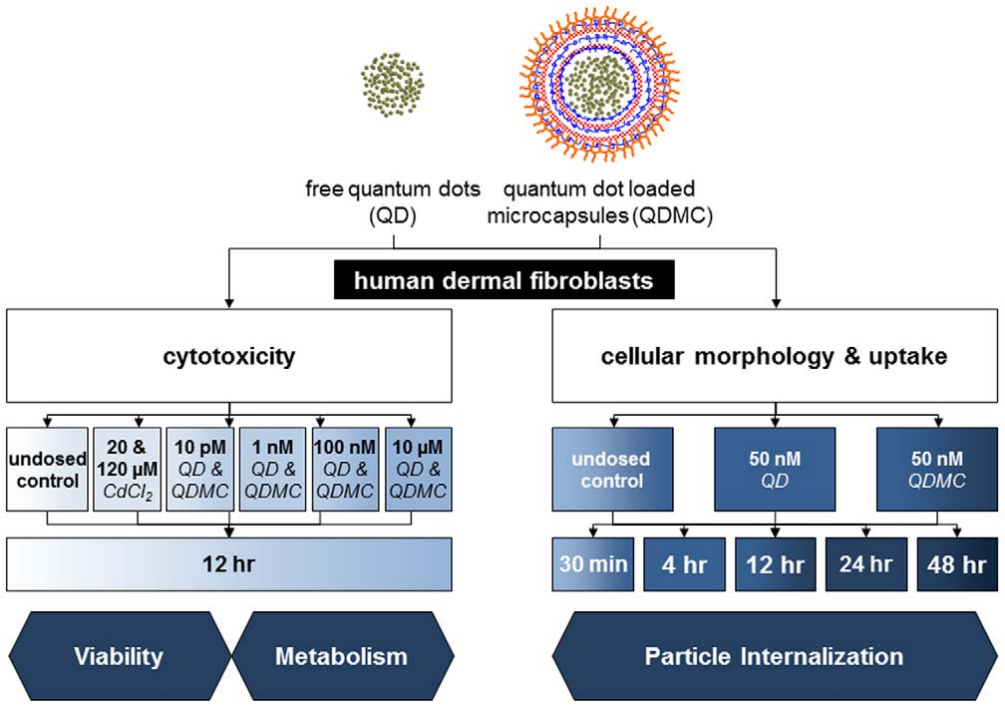
Experimental design. Flowchart of the protocol used for testing the cellular response (cytotoxicity and morphology) of human dermal fibroblasts (HDF) to free quantum dots (QD) and quantum dot loaded microcapsules (QDMC). Cultured cells were exposed to either QD or QDMC for all experiments. CdCl_2_ was used in some experiments as a positive control. Cytotoxicity, including % dead, metabolism, and apoptosis, were determined over a dose-response, while cellular morphology and uptake were determined over a time course.

To more thoroughly mitigate the potential cellular toxicity caused by reported dissociated metal ions [29] or ROS generation from the QDs, we set out to microencapsulate CdSe/ZnS-PEI in an effort to nullify the adverse effects of free QDs on cellular morphology and metabolism, cytotoxicity, and apoptotic or necrotic response (Figure 2). Specific to the case of implantable biosensors under epidermal layers of skin, we have developed a model that investigates the potential cytotoxicity, uptake, and apoptotic response of QD-loaded microcapsules (QDMC). Human dermal fibroblasts (HDF), the cells that compose the majority of the viable dermal layer, were exposed to CdSe/ZnS-PEI QDs microencapsulated within PEG-terminated microcapsules comprised of poly(styrene sulfonate) (PSS) and poly(allylamine hydrochloride) (PAH). The near-neutral surface charge of PEG-terminated microcapsules results in minimized contact with cells, providing an excellent biomedical tool, while retaining desirable QD qualities. However, it is important to note that microencapsulating other types of nanoparticles is not only possible, but potentially advantageous. For example, noble metal nanoparticles that possess antioxidative effects (e.g., reactive oxygen species scavengers) might benefit from microencapsulation to prevent cellular internalization, while simultaneously preserving surface-dependent biomimetic properties. Another potential application is the controlled release of a drug from polymeric nanoparticles, wherein the drug to be delivered acts on cell surface receptors and must be delivered extracellularly (e.g., vascular endothelial growth factor).

## Materials and Methods

Core/shell (CdSe/ZnS) QDs were synthesized in a coordinating solvent tri-n-octylphosphine oxide (TOPO, 99%, Aldrich) in accordance with previously published procedures [30]. The synthesis was performed in a single mode CEM Discover® microwave reactor operating at 300 W, 2.45 GHz. Cadmium oxide (CdO, 99.99%, Alfa Aesar, 0.0514 g, 0.4 mM) along with tetradecylphosphonic acid (TDPA,98%, Alfa Aesar, 0.2232 g, 0.8 mM) and TOPO (3.7768, 9 mM) were heated with continuous stirring in a 125 mL glass flask. The mixture was heated to approximately 300°C under argon (Ar) flow for 15 min. A selenium stock solution (0.0411 g, 0.5 mM, Aldrich, 99%) dissolved in 2.4 mL (2 g) of tri-n-octylphosphine (TOP, 99%, Aldrich) TOP) was injected at 270°C and QDs were allowed to grow for 150 s. A ZnS shell was grown on the CdSe cores by injecting a mixture of Zn and S precursors: 1.6 mL (12 mM) dimethylzinc (DMZ-1M in heptane, Aldrich), 0.42 mL (2 mM) hexamethyldisilathiane (HMDS, Aldrich), and 6.3 mL (14 mM) TOP. The reaction mixture was heated for 30 min at 200°C. The quantum yield of the QDs increased on annealing the particles at a temperature of ∼100°C for a period of two hours.

The QDs were surface modified with high molecular weight branched PEI (b-PEI) (Aldrich, MW 25,000) using similarly reported procedures [31]. Briefly, a 10 mg/mL solution of b-PEI in chloroform was mixed with an equal volume of 4–5 µM QDs. The mixture was tumbled overnight at room temperature. QDs were precipitated from the mixture by addition of excess cyclohexane (Sigma-Aldrich, >99%) and suspended in deionized water. Excess PEI was extracted from the aqueous QD solution by addition of fresh chloroform, which phase-separated from water. The zeta potential of the PEI coated QDs was measured at approximately +29.7±6.2 mV by a Zetasizer Nano ZS (Malvern Corp, Worcestershire, UK). The quantum yield of PEI coated QDs as produced is estimated to be 11.1%.

To microencapsulate the QDs in microcapsules (Figure 1), QDs were first entrapped in CaCO_3_ microparticles using a modification of Petrov's protocol [32]. Briefly, 2.4 mL of a 2.17 µM PEI-coated QD solution was added to 7.6 mL of a Na_2_CO_3_ solution containing 40 mg of PSS (M_w_ = 70 kDa) for a final Na_2_CO_3_ concentration of 0.2 M. While stirring the Na_2_CO_3_ solution, 10 mL of a 0.2 M CaCl_2_ solution was quickly added. After stirring for 30 s, the resulting particle suspension was centrifuged at 2500 *g* for 5 min to remove any remaining free QDs and unreacted salts; the precipitate was rinsed three times with deionized water and imaged using an inverted epifluorescence microscope (Nikon Eclipse TE2000-U) with a 40× objective (Nikon Plan Fluor, 0.75), 1.5× secondary magnification, and a color digital camera (Nikon DS-Fi1). Particle size measurements were performed using an Elzone II Particle Size Analyzer equipped with a 30 µm orifice tube (Micromeritics, Norcross, GA). Particle diameter was validated via optical microscopy. Zeta potential measurements, as an indicator of microcapsule surface charge, were performed using a Zetasizer Nano ZS (Malvern Corp, Worcestershire, UK). Encapsulation efficiency was estimated by measuring the fluorescence intensity of the QDMC solution and dividing by the sum of the fluorescence intensities of the QDMC solution and the supernatant solution recovered from the CaCO3 microparticle precipitation, which contained unencapsulated QDs. Due to the high scattering of the samples, a PC1 photon counting spectrofluorometer (ISS, Champaign, IL) was modified using a bifurcated fiber optic bundle to collect fluorescence using 180° collection geometry. Empty CaCO_3_ microcapsules (containing no QDs) were prepared using the aforementioned protocol, with the exception that 2.4 mL of deionized water was substituted for the PEI-coated QD solution.

Nanofilms were deposited on the CaCO_3_ template using the LbL method, in which substrates are immersed in polyelectrolyte solutions of alternating charge [33], [34], [35]. The first ten bilayers were comprised of polyelectrolytes PSS and PAH (M_w_ = 52 kDa). The surface charge of CaCO_3_ microparticles at or below pH 8 is positive, so anionic PSS was the first polyelectrolyte deposited, followed by cationic PAH [36]. Deposition of each polyelectrolyte layer was achieved by suspending the particles in 1 mL of a 2 mg/mL polyelectrolyte solution containing 0.2 M NaCl. Lastly, the particles were coated with a terminal bilayer comprised of PSS and poly-L-lysine(100)-g[4.5]-polyethylene glycol(114) (PLL-g-PEG, Alamanda Polymers, M_w_ = 129 kDa), yielding CaCO_3_ microparticles coated with a (PSS/PAH)_10_/(PSS/PLL-g-PEG) nanofilm. Dissolution of the CaCO_3_ template was accomplished via a mild treatment with 0.1 M ethylenediaminetetraacetic acid (EDTA) adjusted to pH 7.6; complete core dissolution was visually confirmed using phase microscopy.

For viability and internalization experiments, HDF cells (ATCC, Manassas, VA) were cultured in Dulbecco's Modified Eagle's Medium (DMEM, Gibco, Austria), which was supplemented with 10% FBS and an antibiotic cocktail consisting of penicillin, streptomycin, and amphotericin (Sigma-Aldrich, St. Louis, MO). Incubation took place at 37°C with humidity and 5% CO_2_.

Live cells were cultured as above and treated with 50 pM QD and QDMC for 30 min, 4 hrs, 12 hrs, 24 hrs, and 48 hrs to determine the manner and extent of QD uptake into the fibroblasts. An undosed control was also included for comparison. Then, 0.25 µL stock solution Syto 63 (Invitrogen, Carlsbad, CA) was directly added to 1 mL of media in the borosilicate, dual-well chamber slides (Lab-Tek, Nagle Nunc Intl., Rochester, NY) for a 30 min incubation at 37°C. A Zeiss 510 Meta confocal microscope was utilized at 40× (1.3 NA, oil immersion) to image the treated and stained live cells. A 488 nm Ar ion and a 633 nm He-Ne laser were employed to excite the QDs and stain, respectively. Zeiss LSM 510 Software was used to acquire the images.

Cell populations (n = 4) were then seeded in 24 well plates, allowed to grow to 80% confluency, and dosed with 0.05–50 nM concentration range for both the QD and QDMC or left undosed as a negative control. A 20 and 120 µM CdCl_2_ solution was utilized as a positive indicator for absence of fluorescent probe metabolism. The dosed media was aspirated after 12, 24, or 48 hr. The cells were washed with PBS and phenol red-free DMEM media was added. Resazurin dye (10% v/v, Sigma Aldrich, St. Louis, MO) was added and the cells were incubated for an additional 3 hr. Fluorescence intensity was obtained by exciting at 560 nm and recording emission at 590 nm. Fluorescence compatibility was measured prior to assay (Figure S2). QD and QDMC toxicity to cells was measured in terms of percent healthy cells via microscopy.

## Results

The average microparticle diameter was determined to be 5.24 µm as measured with a particle size analyzer, with a standard deviation of 1.25 µm (95% confidence interval: 5.165–5.317 µm) (Table 1 and Figure 1B). This diameter was also observed via optical microscopy. The encapsulation efficiency was determined to be ∼30.57%, resulting in about 9,000,000 QDs/microcapsule. At this encapsulated concentration, the volume occupied by the microencapsulated QDs is ∼0.1% of the total microcapsule interior volume. Based on the diameter of the microcapsule, the average surface area would be 7.854×10^−11^ m^2^ (i.e., 78.54 µm^2^).

**Table 1:** QD and QDMC characterization table.

Property	QD	QDMC
Size in water	14–16 nm	3–5 µm
Surface charge	+29.7±6.2 mV	near neutral*
Interior volume	-	14.14–65.45 µm^3^
Calculated surface area	∼706 nm^2^	28.27–78.54 µm^2^
No. of QDs/microcapsule	-	2.05e10
No. of Cd^+2^ ions/system	265.8	5.46e12
Excitation/Emission	380/545 nm	380/545 nm

*As measured on CaCO_3_ microparticles coated with (PSS/PAH)_10_/(PSS/PLL-g-PEG) nanofilms.

Figure 1 depicts the process used to form the QDMC. Morphology of the microcapsules was determined with microscopy. Following core dissolution, brightfield and fluorescence images of the resulting microcapsules were acquired (Figure 1 C&D). The brightfield image reveals a spherical morphology, and the fluorescence image confirms successful QD microencapsulation. Both the micrographs and the histogram show that the QDMC sample contains monodisperse microcapsules. In addition, QDs do not show evidence of self-quenching (i.e., red-shift of emission peak) once microencapsulated, as shown by the fluorescence emission spectra in Figure S3.

**Figure 3:**
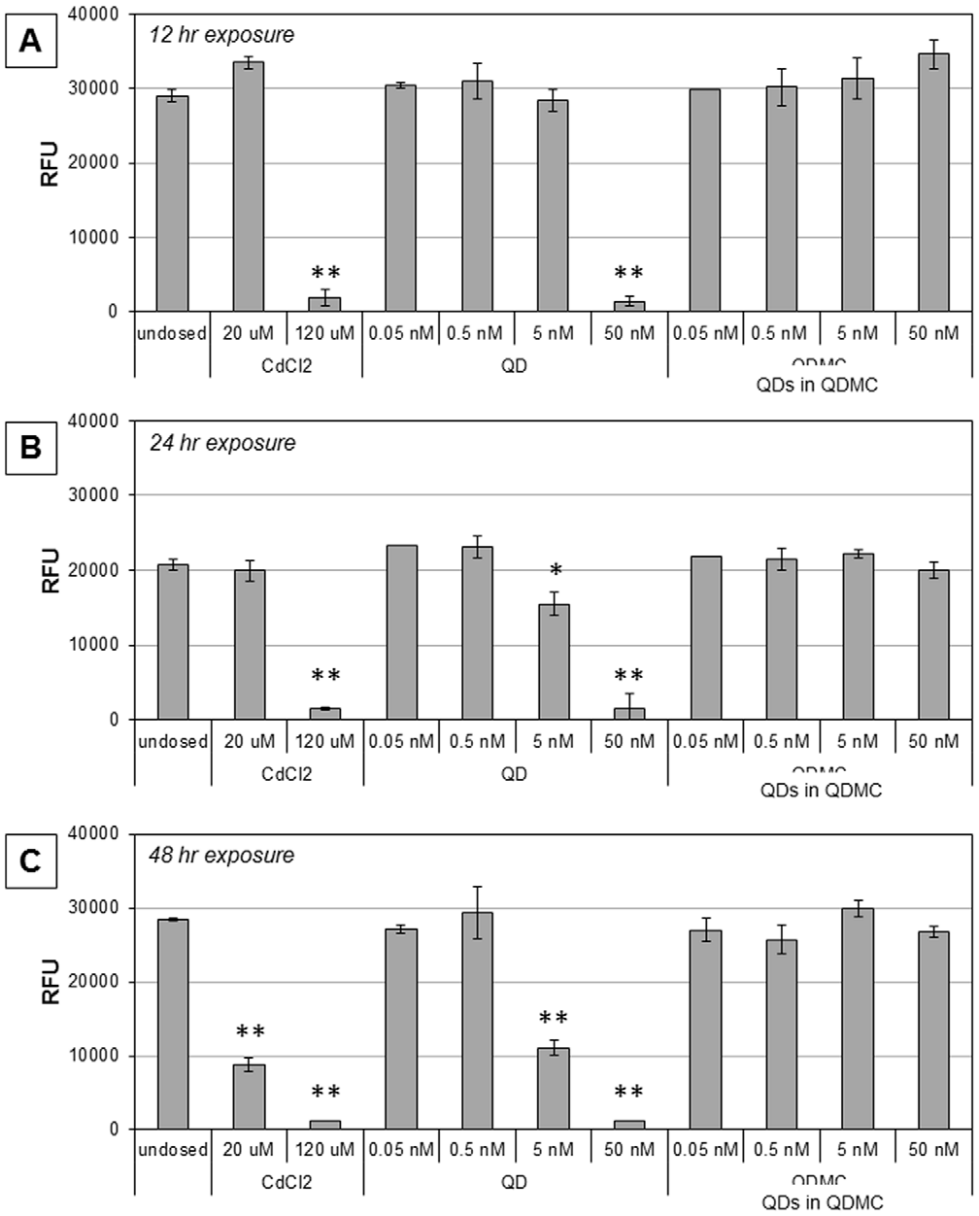
Differential cytotoxicity. Cytotoxicity was measured at A) 12 hrs, B) 24 hrs, and C) 48 hrs via fluorescence intensity of reduced resazurin. HDF cells at all time points were either exposed to 0.05–50 nM QDs or microencapsulated QDMCs, 20 or 120 µM cadmium chloride positive control, or unexposed. * p-val<0.05, ** p-val<0.001. Note: the QDMC concentrations refer to the total concentration of QDs contained in the microcapsules.

**Figure 4:**
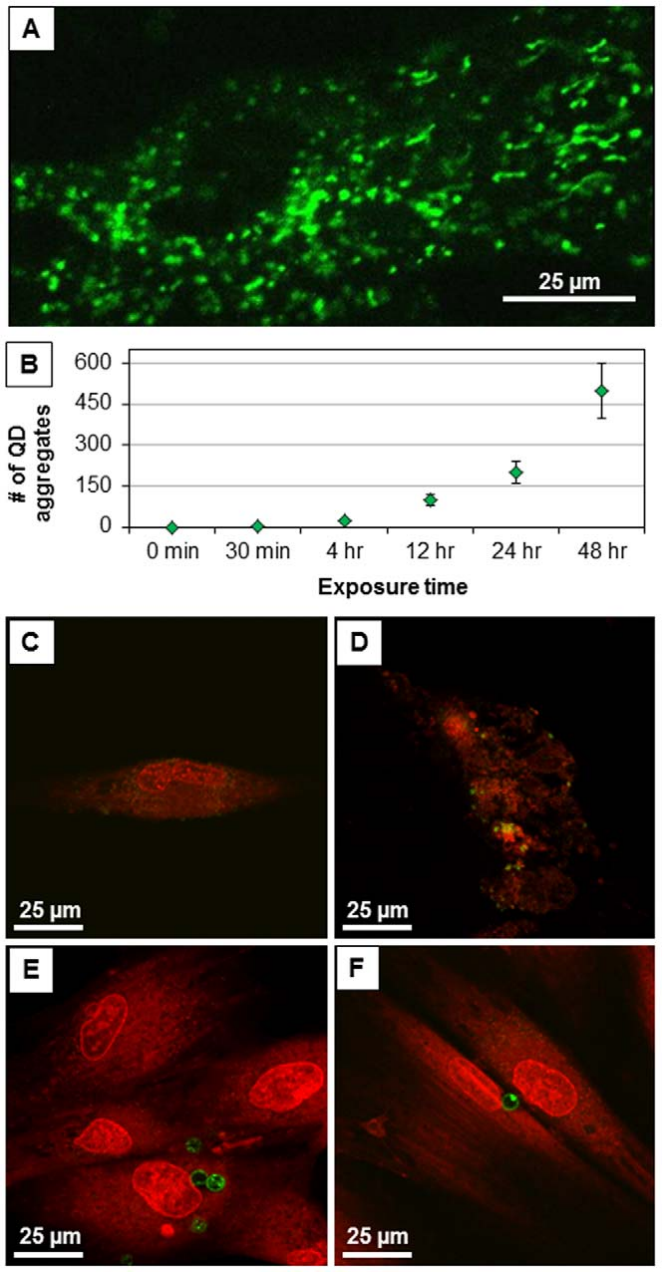
QD association and cellular morphology. (A) QD accumulation in HDF mitochondria (Confocal, 60×, Zeiss). (B) The number of QD aggregates accumulating in HDF increases over time. Fluorescence images of cells dosed with QDs at the (C) 12 hr and (D) 48 hr time points. Images of cells dosed with QDMCs (at an equivalent amount of Cd^+2^ ions relative to QDs) at the (E) 12 hr and (F) 48 hr time points. Microcapsules are resting on top of cells, but do not enter cells. Cells dosed with an equivalent Cd atom concentration of the QD-loaded microcapsules displayed no evidence of particle uptake and remained viable with no sign of deterioration through the 48 hr time point.

Cellular viability was measured in two independent studies. First, viable cells were counted using brightfield microscopy (data not shown). Second, changes in metabolic activity were measured using the conversion of resazurin to resorufin (Figure 3). In both studies, HDF cells were exposed to QDs, QDMCs, and CdCl_2_ (positive control). For comparison, the total number of QDs at each concentration was kept constant between the QD and QDMC samples; e.g., 5 nM QDMC refers to enough MCs to provide a total QD concentration of 5 nM. All responses were compared to those of undosed cells. Results indicated a strong dose- and time-dependent response relationship present in QD and CdCl_2_-treated cells, but no significant changes in metabolic activity were found by 48 hr in QDMC-treated cells. Cells exposed to empty microcapsules were also examined for changes in viability and metabolic activity and no significant changes were found. These findings corroborate the cellular morphology microscopy data (Figure 4).

It is important to note that QDs are similarly toxic at much lower concentrations than the cadmium chloride positive control. This is not surprising since there are factors other than chemical composition (e.g. surface charge, particle size, ROS generation potential) that must be considered when evaluating nanoparticle cytotoxicity. For example, it has been shown that cadmium-containing QDs are more toxic to bacteria than an equivalent cadmium concentration of cadmium salt due to higher ROS generation, damaged cell membranes, and the presence of Se^0^ and dissolved cadmium [37].

Figure 4 and Figure S1 show that the free QDs visibly disrupted the cellular morphology, which became apparent after the 12 hr time point and progressed throughout the remainder of the experiment. Cells dosed with an equivalent Cd atom concentration of the QD-loaded microcapsules displayed no evidence of particle uptake and remained viable with no sign of deterioration through the 48 hr time point.

## Discussion

Due to the recent surge of nanoparticle development for imaging and therapeutic applications, there is reason to evaluate toxicity of nanomaterials. Some studies suggest nanoparticles do affect biological systems at the cellular, subcellular, and protein levels [38], [39], [40]. Other works indicate that nanoparticles are cleared from circulation by macrophages depending on particle composition, size, charge, surrounding pH, dose, or route of exposure [41], [42], [43], [44]. Nevertheless, there are concerns that Cd-containing QDs are toxic to both cell cultures and live animals because they contain a toxic heavy metal. Are there engineering solutions that can mitigate the nanoparticle toxicity while maintaining functionality?

Previous studies have shown that the endocytosis of nanoparticles in acidic endosomes degrade nanomaterials and cause leaching of metals, thus producing stress, triggering apoptotic response, and eventual death [45]. We propose that the observed death could be mitigated by controlling uptake via microencapsulation and altering microcapsule outer surface charge. These mitigations can improve the biocompatibility of QDs by decreasing internalization. Data from the literature has indicated that particles that remain in the extracellular matrix are typically less toxic to cells than particles that have breached the cytoplasmic membrane [46], [47], [48]. Research presented in this paper suggests that QDMC biocompatibility is due to lack of cellular uptake and is evidenced by normal mitochondrial function. Therefore, as new nanomaterials (including microencapsulated systems or composited materials) that possess useful physical and chemical properties are developed, understanding the propensity for cellular uptake and subsequent adverse cellular effects is vital.

Cellular uptake is an additional parameter to be considered in the design and evaluation of biocompatible nanoparticles for biological applications. This data provides strong evidence that specific microencapsulation of CdSe/ZnS particles imparts substantial protection. The most common capping strategies are known to decrease toxicity, but they also often decrease functionality by increasing the size of the QDs. We have shown here that by microencapsulation of the nanoparticle, toxicity is decreased, while luminescence – a precursor for functionality – remains uncompromised.

## Supporting Information

Figure S1 **Cellular morphology over time.** Micrographs B through F show the cellular morphology of HDFs exposed to QDs over a 30 minute to 48 hour time course study. As exposure time increases, the cells exhibit compromised cytoplasmic membrane. Micrographs H through L show the cellular morphology of HDFs exposed to QDMC over a 30 minute to 48 hour time course study. Cells remain intact and healthy over the course of the 2-day study. Z-stack images indicate that microcapsules do not enter cells.
(TIF)

Figure S2 **Fluorescence compatibility of QDs and resorufin in resazurin assay.** Fluorescence spectral emission wavelengths of QDs and resorufin, product of resazurin, when excited at 560 nm. Resazurin assay data was collected at 590 nm.
(TIF)

Figure S3 **Unencapsulated QDs and microencapsulated QDs fluorescence emission spectra (after normalization to the peak intensity).** Because the spectra are identicalshow no spectral shift to longer wavelength upon microencapsulation, the quantum dotQDs are not self-quenching within the microcapsules after microencapsulation. The spectra are essentially the same, and there is no evidence of the characteristic red-shift that is associated with self-quenching.
(TIF)
