# Supplementary figures and images for: Mitigation of Quantum Dot Cytotoxicity by Microencapsulation
Source: PLoS One. 2011 Jul 21;6(7):e22079. doi: 10.1371/journal.pone.0022079 (PMC3140988; doi:10.1371/journal.pone.0022079)

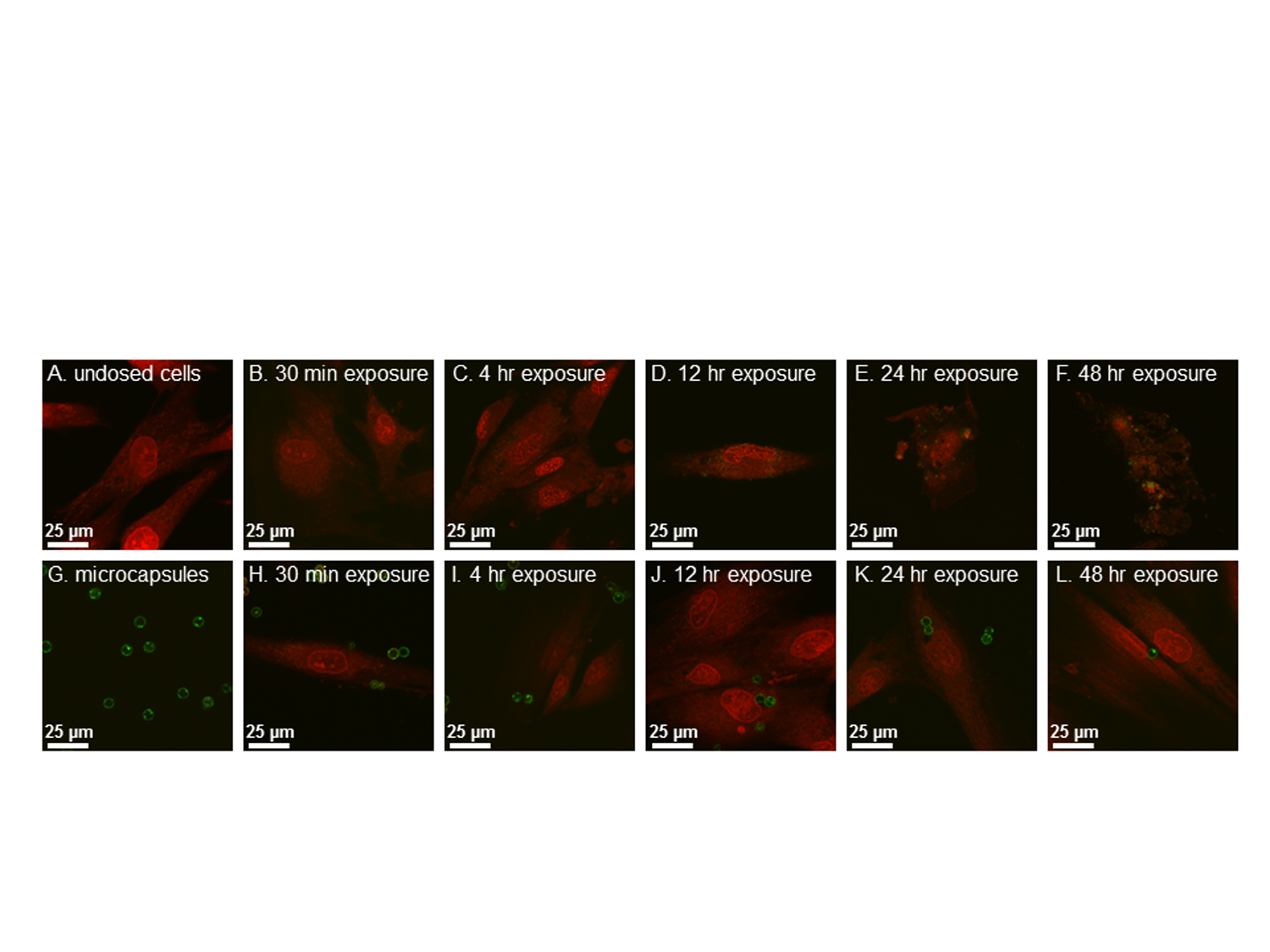

Supplement: Figure S1 — Cellular morphology over time. Micrographs B through F show the cellular morphology of HDFs exposed to QDs over a 30 minute to 48 hour time course study. As exposure time increases, the cells exhibit compromised cytoplasmic membrane. Micrographs H through L show the cellular morphology of HDFs exposed to QDMC over a 30 minute to 48 hour time course study. Cells remain intact and healthy over the course of the 2-day study. Z-stack images indicate that microcapsules do not enter cells. (TIF) [file pone.0022079.s001.tif]

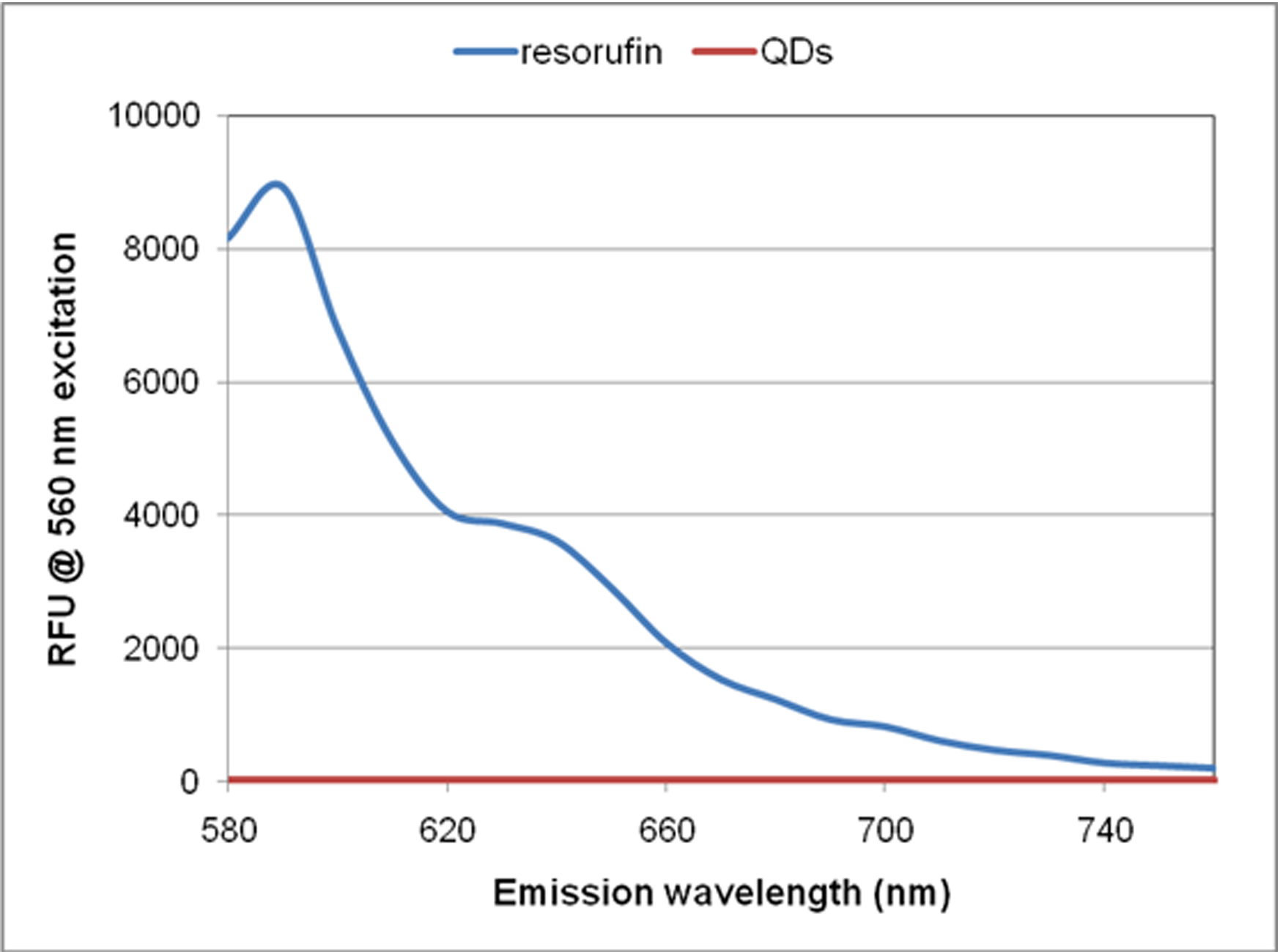

Supplement: Figure S2 — Fluorescence compatibility of QDs and resorufin in resazurin assay. Fluorescence spectral emission wavelengths of QDs and resorufin, product of resazurin, when excited at 560 nm. Resazurin assay data was collected at 590 nm. (TIF) [file pone.0022079.s002.tif]

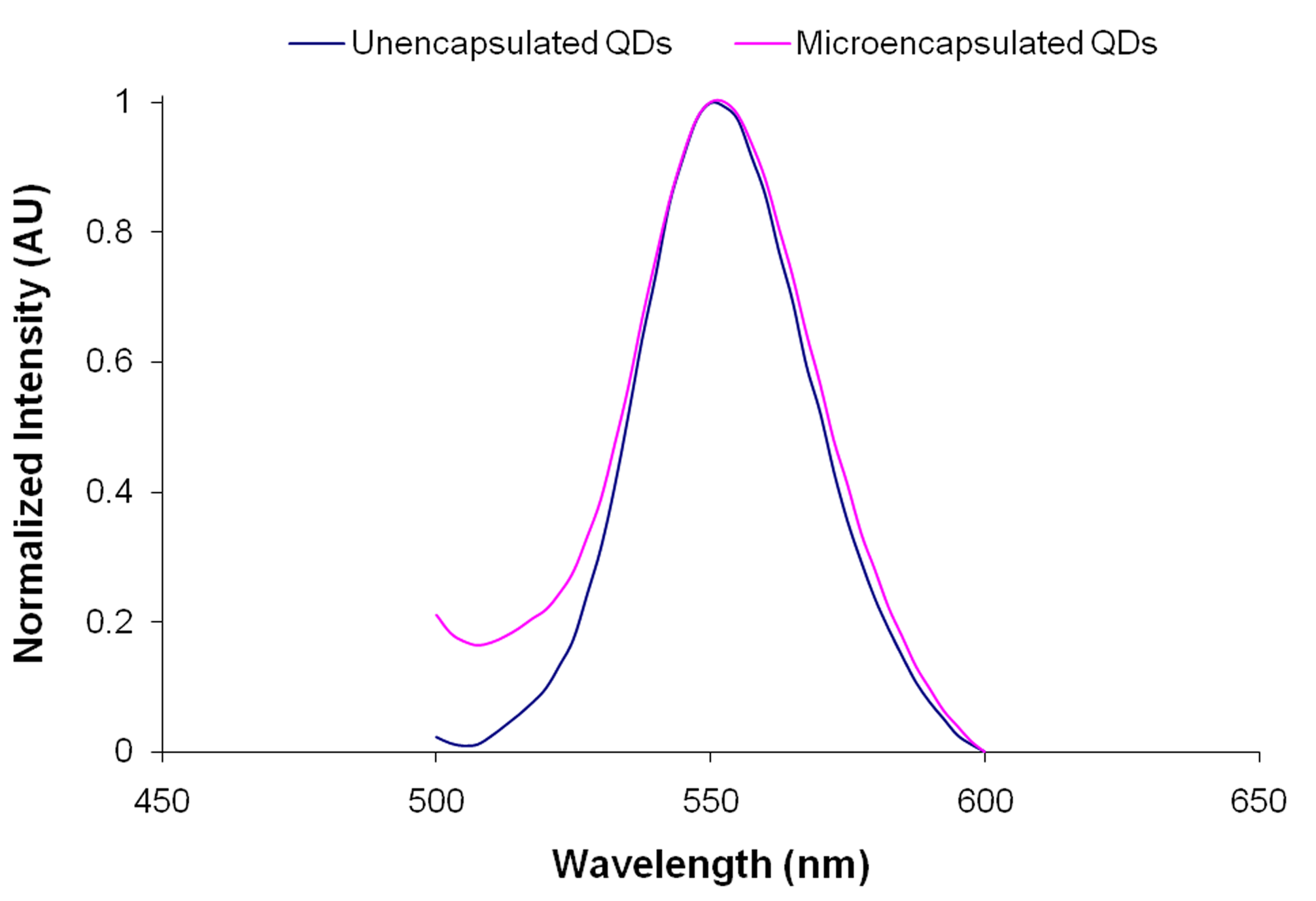

Supplement: Figure S3 — Unencapsulated QDs and microencapsulated QDs fluorescence emission spectra (after normalization to the peak intensity). Because the spectra are identicalshow no spectral shift to longer wavelength upon microencapsulation, the quantum dotQDs are not self-quenching within the microcapsules after microencapsulation. The spectra are essentially the same, and there is no evidence of the characteristic red-shift that is associated with self-quenching. (TIF) [file pone.0022079.s003.tif]
